# Visual outcomes of lens subluxation surgery with Cionni modified capsular tension rings in Marfan syndrome

**DOI:** 10.1038/s41598-021-82586-6

**Published:** 2021-02-04

**Authors:** Tianhui Chen, Michael Deng, Min Zhang, Jiahui Chen, Zexu Chen, Yongxiang Jiang

**Affiliations:** 1 Department of Ophthalmology and Vision Science, Eye and ENT Hospital of Fudan University, 83 Fenyang Rd, Shanghai, 200031 China; 2 Key Laboratory of Myopia of State Health Ministry, and Key Laboratory of Visual Impairment and Restoration of Shanghai, Shanghai, China

**Keywords:** Lens diseases, Risk factors, Eye manifestations

## Abstract

Marfan syndrome (MFS) is a hereditary disease with an incidence of 0.3% in the general population. Approximately 60% of MFS patients with FBN1 gene mutation will suffer ectopia lentis (EL) from the age of 3. With the development of EL, severe loss of vision will accrue because of lens tilt and glaucoma. Cionni modified capsular tension rings (MCTR) has been applied in the surgery for EL in MFS patients. To evaluate visual acuity and safety of using MCTR during lens subluxation surgery in MFS patients, 66 MFS patients (110 eyes) were included in our study, with the mean duration of follow-up of 4.7 months (SD 1.76 months). The capsular bags were preserved in 101 eyes (91.81%) with MCTR implantation. There was an overall significant improvement in BCVA at 1-month follow-up which was maintained at 3 months. Multivariable linear regression revealed that older age at first visit was associated with greater postoperative BCVA at the 1-month follow-up (*P* = 0.007). A significant difference was found between different degrees of lens subluxation and the length of surgical time and complications. At follow-up, only two eyes (1.98%) were identified to have developed retinal detachments. In conclusion, better visual outcomes can be achieved when patients received an early operation with MCTR implantation.

## Introduction

Marfan syndrome (MFS) is an autosomal dominant disorder affecting several systemic systems, such as the cardiovascular and ophthalmologic system. The prevalence has been estimated to be 1 in 10,000 to 20,000 individuals without geographic, ethnic, or gender predilection^1,2^. The mutation of the fibrillin-1 (FBN-1) gene located on chromosome 15, which is the main component of elastic matrix microfibrils is acknowledged as the main cause of MFS with a detection rate of 97% among MFS patients^1,3,4^.

Affected individuals present with typical manifestations of MFS during all phases of life, including descending aortic root aneurysm and ectopia lentis (EL). EL occurs in approximately 60% of MFS patients^5,6^. Maumenee et al. ^7^ reported that EL was stably accrued in 12.5% of MFS children before the age of 3 and 45% of those at the age of 4 to 5, which could be the initial presenting sign of MFS. Other well-recognized ocular manifestations are myopia, early cataract, glaucoma, and retinal detachment^5,8,9^.

Without adequate zonular support for the lens, EL may cause a large refractive error, a partially phakic visual axis, and an anisometropia. Furthermore, the risk of developing glaucomatous damage, secondary microspherophakia, as well as endothelial compromise, can also be highly ascended by severe EL^10^. The lack of FBN-1 in the capsular bags and zonules as well as in the iris and sclera of MFS patients, which are the two ideal positions for IOL fixation, results in difficulty in the fixation of Scleral- and iris-fixated intraocular lens (IOL)^11,12^. As a consequence, intra-operative and postoperative complications have been reported^12,13^, including iris capture and lens decentration due to deficient FBN-1 in the iris and sclera. These pathological changes make EL in the setting of MFS to be one of the most challenging anterior segment surgeries.

The transscleral suture-fixated IOL implantation has previously been accepted as an ideal operative method for the surgical correction of EL. However, it is associated with a high risk of serious complications such as retinal detachment (RD) or glaucoma^11^. Moreover, intra-operatively during this procedure, the needle needs to be passed through the vascular uveal tissue that may result in bleeding and great trauma^14^. From a survey participated by 185 MFS patients with EL and RD, 21% with RD had prior lens surgery^11^. Furthermore, at 7–10 years after transscleral suture-fixated IOL implantation, IOL dislocation has been observed in 24% of patients due to breakage in the polypropylene suture^14^.

When performing anterior segment procedures, preservation of capsular bags by implanting the Cionni modified capsular tension rings (MCTR) has become a preferred practice instead of transscleral fixated IOL implantation for MFS patients^15^. MCTR can provide adequate support and correct the decentration of capsular bags in the presence of progressive zonular degradation. As a simplified operation, MCTR can be implanted into capsule bags with greater ease and minimal trauma. It can also be easily fixated to the sclera with 1 or 2 sutures without compromising the integrity of the capsular bag. Other advantages of MCTR implantation, such as minimizing vitreous loss and allowing placement of posterior chamber IOL (PC-IOL) in capsular bags have also been reported^16^. Furthermore, the long-term risk of postoperative complications including retinal detachment, glaucoma, and IOL dislocation can also be reduced by implanting MCTR in eyes with subluxation of the lens secondary to MFS^15,17^. However, the evidence is still lacking in the practice of MCTR implantation for the subluxation of the lens with its visual outcomes in the MFS cohort.

In this study, we aimed to evaluate the visual outcomes of MFS patients following MCTR implantation in eyes with a subluxated lens. Length of surgical time, complications of the surgery, and predictive factors for postoperative best-corrected visual acuity (BCVA) were also investigated.

## Materials and methods

### Participants

**Figure 1:**
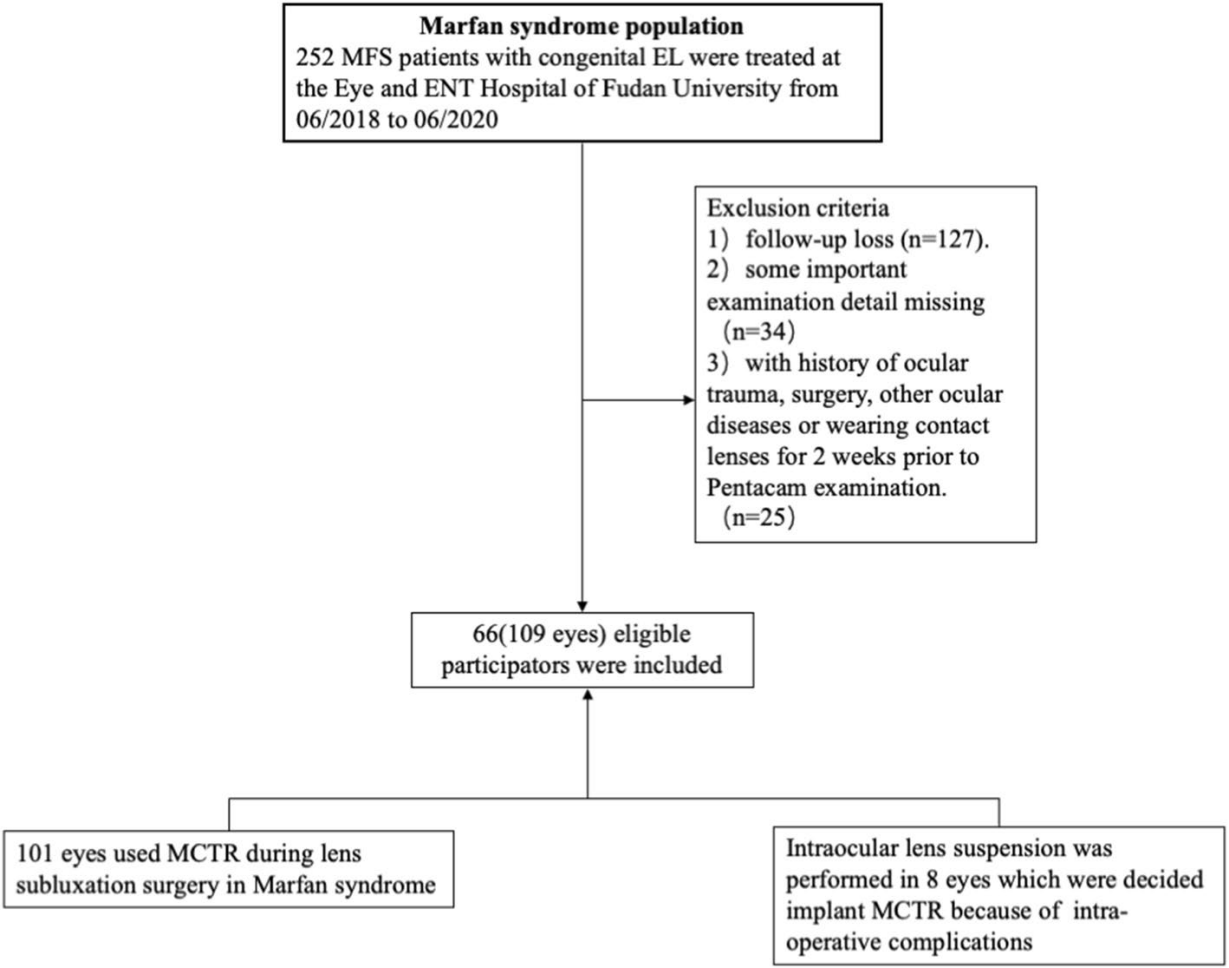
Flow chart illustrating the selection of patients in this study.

From June 2018 to June 2020, a total of 252 MFS patients with congenital EL at the Eye and ENT Hospital of Fudan University were retrospectively reviewed and screened for suitability for this study. All of these patients had MFS confirmed by Ghent-2 criteria. Patients with microspherophakia, keratoconus, previous history of ocular surgery, uveitis, corneal disease, glaucoma, retinal detachment, or use of contact lenses in the 2 weeks prior to the examinations were excluded from this study. Finally, 66 (110 eyes) MFS patients with EL who were suitable for surgical intervention were included in this study. The family and medical histories of all patients were carefully recorded. A flow chart summarizing the selection of the study cohort was illustrated in Fig. 1.

### Ethics statement

This study was approved by the Human Research Ethics Committee of the Eye and ENT Hospital of Fudan University that adhered to the tenets of the Declaration of Helsinki. All the participants have given their written informed consent and informed consent for the publication of the images and data, of which the participants under the age of 18 years were provided through their legal guardians. This study was an extension of our randomized controlled trial (ChiCTR2000039132).

### Data collected

In accordance with previously published reports, data on patient demographics, preoperative ocular parameters measured by Pentacam AXL system, best-corrected visual acuity (BCVA) during the first visit and 1-, 3-, and 6-month postoperative follow-ups, degree of lens subluxation, and surgery time were collected. Intra-operative and postoperative complications were recorded, including iris dysgenesis, vitreous prolapse, a peripheral extension of the tear, retinal detachment, posterior capsular opacification (PCO), and anterior capsule opacification (ACO). The logarithm of the minimum angle of resolution (logMAR) units was used to describe BCVA.

**Figure 2:**
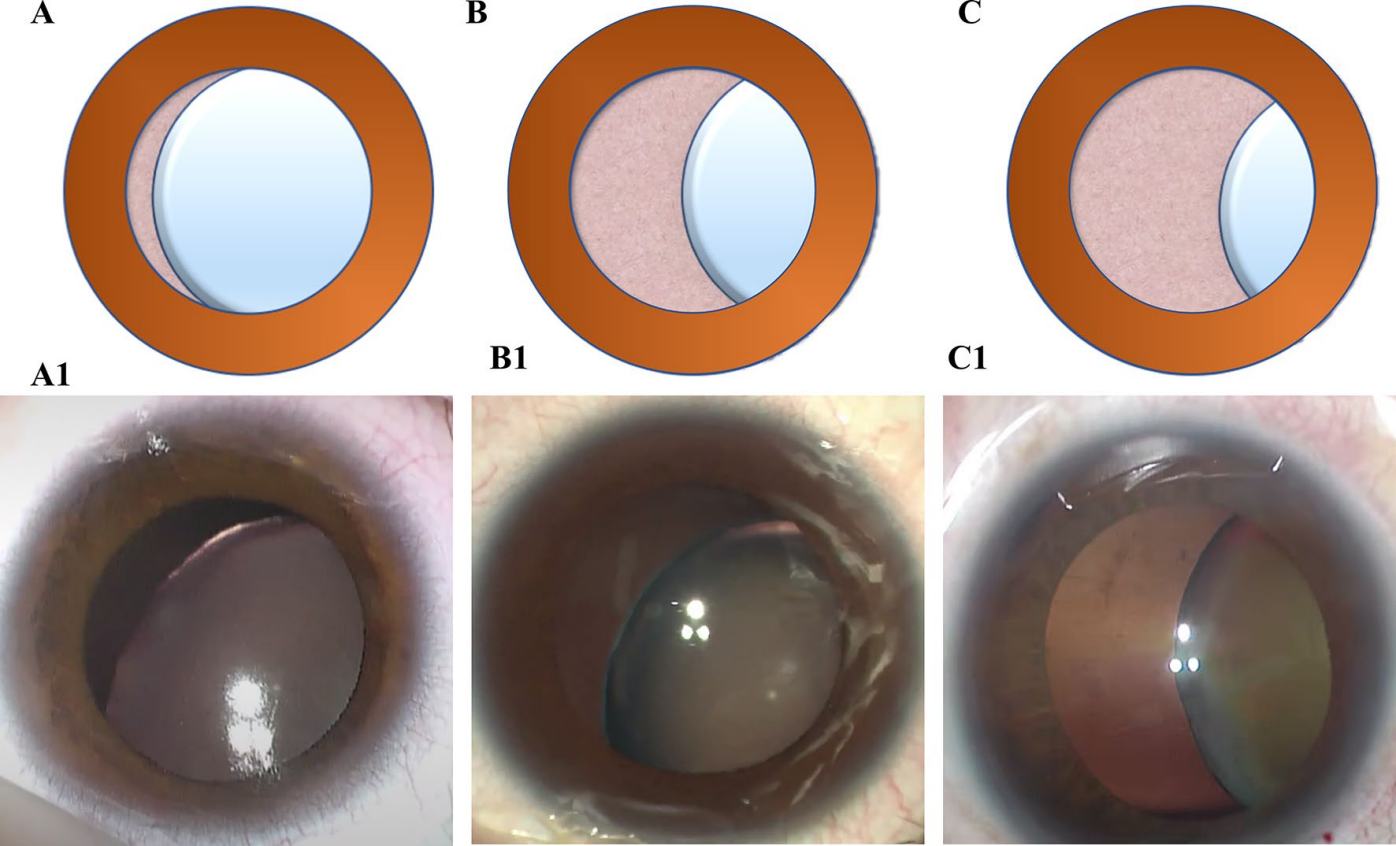
Different degrees of lens subluxation. (**A**) and (**A1**). Mild: lens edge uncovered 0% to 25% of the dilated pupil; (**B**) and (**B1**). Moderate: lens edge uncovered 25% to 50% of the dilated pupil; (**C**) and (**C1**). Severe: lens edge uncovered greater than 50% of the dilated pupil. All images were taken during the operations by Doctor YX Jiang. Written informed consent was obtained from all the participants.

For analyses, eyes were divided into 3 groups based on the BCVA: Group 1: logMAR < 0.3 (Snellen equivalent 20/40); group 2: 0.3 < logMAR < 1 (Snellen equivalent between 20/200 and 20/40); group 3: logMAR > 1 (Snellen equivalent 20/200 or worse). The degrees of lens subluxation were stratified into 3 broad groups (**Fig. ****2**): mild: lens edge uncovered 0% to 25% of the dilated pupil; moderate: lens edge uncovered 25% to 50% of the dilated pupil; severe: lens edge uncovered greater than 50% of the dilated pupil.

The Cataract Pre OP pattern of the Pentacam AXL system (Oculus Inc., Wetzlar, Germany) with a rotating Scheimpflug camera was used to measure the biological characters of the eyes in MFS patients.

All patients were examined by experienced ophthalmologists with data recorded as the means of three repeated measurements.

### Surgical technique

All surgeries of scleral-fixated MCTR implantation with intraocular lenses (IOL) were performed by one surgeon (Dr. YX Jiang). A 2.6 mm clear corneal incision was made after general anesthesia. A continuous curvilinear capsulorrhexis was created. To stabilize the capsular bag, 2–4 capsular hooks (Madhu Instrument Pvt. Ltd, India) were applied. Soft nucleus and cortical aspiration were performed using an irrigation/aspiration handpiece (Alcon Laboratories Inc.) under a low flow rate with an infusion bottle height of 65 cm. After the capsular bag was refilled with DisCoVisc (Alcon Laboratories, Ft Worth, Tex), MCTR was positioned in the area of maximum zonular weakness with the fixation eyelet through the main incision into the expanded capsular bag. The MCTR was sutured with 9–0 polypropylene to the sclera, 1.5 mm posterior to the corneal limbus. The suture needle was passed around the interlamellar sclera four times with a modified knotless Z-suture technique. The suture was tightened and cut. The end of the suture was spontaneously retracted to the interlamellar sclera. Then, an acrylic 1-piece foldable IOL (Tecnis: ZCB00; Alcon: AcrySofSN60WF or SN60AT) selected by the surgeon was implanted into the capsular bag. After aspirating residual OVD by I/A handpiece, the main corneal wound was sutured with 10–0 nylon suture while the overlying conjunctiva was closed with 8–0 vicryl suture.

Postoperatively, Cravit Eye Drops (Santen Pharmaceuticals, Inc., Osaka, Japan) and Pred Forte Eye Drops (Allergan Pharmaceuticals, Inc., Dublin, Ireland) were applied three times daily for 1 month. Also, 0.1% pranoprofen (Sumika Finechem, Osaka, Japan) was applied three times daily for 1 month, followed by weekly tapering.

### Statistical analyses

The average standard deviation (SD) was used to describe continuous variables. Categorical variables were described by number and proportion as appropriate. To confirm the normal distribution of the variables, the Kolmogorov–Smirnov test was used. The Chi-square test, Student’s *t*-test, one-way ANOVA, and Mann–Whitney U-test were used for analyzing the data between the modes of different groups.

Univariable and multivariable line regression analyses were performed to identify the predictors of BCVA at 1-month, 3-month, and 6-month postoperatively.

## Results

### Preoperative characteristics

The cohort for this study consisted of 66 (110 eyes) MFS patients with EL. However, transscleral suture-fixated IOL was performed in 9 eyes (8.18%) instead of the originally-planned MCTR implantation because of intra-operative complications. The baseline preoperative ocular characteristics of the study participants (101 eyes) who underwent MCTR implantation were shown in Table 1.

**Table 1:** Baseline characteristics of the preoperative MFS group.

	MFS group
Subjects/eyes	66/101
Sex (female:male)	33:33
Eyes (right:left)	54/47
Age (years)	15.53 ± 13.05
Follow-up time (months)	4.7 ± 1.76
Preoperative BCVA (logMAR)	0.64 ± 0.41
Central ECC (cells/mm2)	3329.11 ± 330.06
Corneal pachymetry (μm)	541.99 ± 46.85
WTW (mm)	12.38 ± 3.24
LT(mm)	3.85 ± 0.69
AL (mm)	24.83 ± 3.15
Km F (D)	40.96 ± 1.69
Astig F (D)	1.98 ± 0.96
Km TCRP (D)	40.6 ± 1.69
Astig TCRP (D)	2.16 ± 1.07
WFA 4-mm zone (D)	− 1.83 ± 1.08
WFA Z40 (D)	0.09 ± 0.1
WFA HO RMS (D)	0.18 ± 0.14
ACD int (mm)	2.78 ± 0.55
B/F ratio	82.7 ± 1.77

BCVA: best-corrected visual acuity; ECC: endothelial cell count; WTW: white to white; LT: lens thickness; AL: Axial length; Km: mean keratometry; F: front (anterior corneal surface); Astig: astigmatism; TCRP: total corneal refractive power; WFA 4-mm zone: wavefront aberration in the 4-mm zone around the corneal apex; WFA Z40: total corneal spherical aberrations (Z4,0) in the 6-mm zone around the corneal apex; WFA HO RMS: root mean square of the total corneal high order aberrations calculated in the 4-mm zone around the corneal apex; B/F ratio: mean radius of the posterior corneal surface/mean radius of the anterior corneal surface ratio; ACD: anterior chamber depth; Cornea: corneal diameter (horizontal).

### Intra-operative complications

After reviewing the surgery video of 103 EL eyes in this cohort (video data of 6 eyes with MCTR implantation was not available), a peripheral extension of the tear occurred in 7 eyes (6.8%), of which 2 developed further to a posterior extension. As a result of the peripheral extension of the tear, MCTR could not be implanted or had to be removed in 6 patients. In preoperative EL, the vitreous loss occurred in 7 eyes (6.8%) with severe zonular weakness, the breakup of the anterior vitreous membrane occurred in 5 eyes, and vitreous prolapse occurred locally in intra-operative traction of capsular bags in 2 eyes. After vitrectomy of 5 eyes, MCTR could still be maintained and IOLs were implanted into capsular bags. Transscleral suture-fixated IOL was performed on 2 eyes with vitreous loss. Iris dysgenesis appeared in 5 eyes (4.85%). Surgery time and intra-operative complications were analyzed according to the different degrees of lens subluxation, as illustrated in Table 2. Significant differences were observed in surgery time, vitreous loss, and peripheral extension of the tear in different degrees of lens subluxation while there was no difference in iris dysgenesis with the varying degrees of lens subluxation.

**Table 2:** Surgery time and intra-operative complications in different degrees of lens subluxation.

	Mild	Moderate	Severe	*P* value
Eyes	39	37	27	NA
Surgery time/m	19.87 ± 2.98	22.33 ± 7.08	31 ± 10.57	< 0.001
Iris dysgenesis/N	2(5.13%)	3(8.1%)	0(0%)	0.073
Vitreous prolapse/N	0(0%)	4(8.11%)	3(11.11%)	0.023
Peripheral extension of the tear/N	1(2.56%)	1(2.7%)	5(18.52%)	0.03

### Post-operative complications

Retinal detachment (RD) occurred in 2 MFS patients (1.94%) postoperatively, first in a 10-year-old girl at 1 month after MCTR implantation, and another occurred in the left eye of a 24-year-old man on the second day after surgery. After detachment surgery and posterior capsulotomy by YAG laser, his BCVA was 20/40 at the 3-month follow-up. PCO was found in 22 eyes (21.36%) and ACO was found in 11 eyes (10.68%) at 6-month follow-ups. Dislocation of IOL-capsular bag complex was observed in 6 eyes (5.94%) with no obvious influence of visual outcome in 4 eyes while anterior capsulotomy by YAG laser was performed in 2 eyes compared with ACO. There were no cases of postoperative endophthalmitis or vitreous in the anterior chamber.

### Visual outcomes

**Figure 3:**
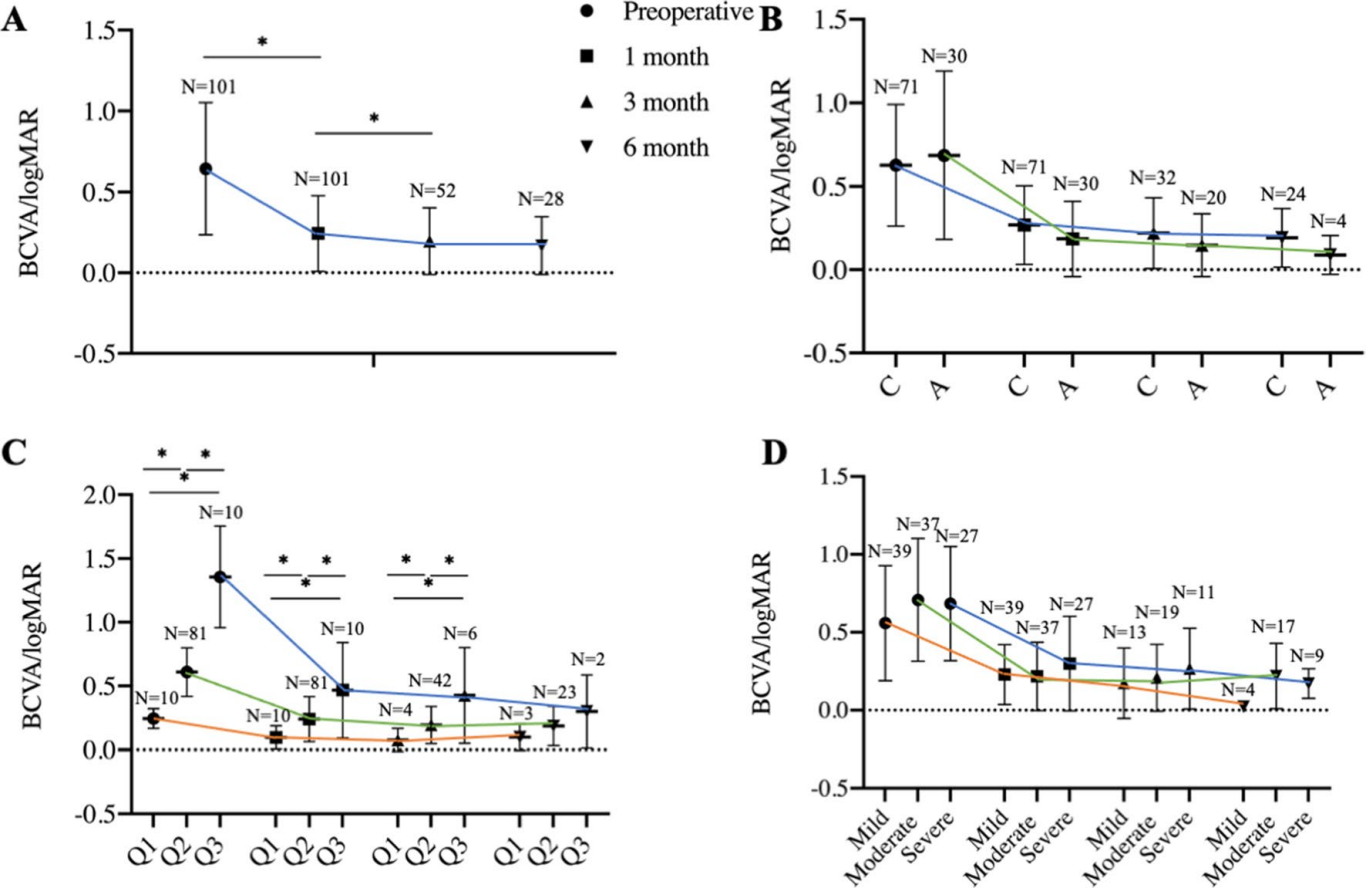
BCVA in eyes that underwent MCTR implantation. (**A**) Changes of BCVA in all patients with MCTR implantation. (**B**) Changes of BCVA in the “children” group and “adult” group. C: Children; A: Adult. (**C**) Changes of BCVA in Q1, Q2, and Q3. Q1: logMAR < 0.3 (Snellen equivalent 20/40); Q2: 0.3 < logMAR < 1 (Snellen equivalent between 20/200 and 20/40); Q3: logMAR > 1 (Snellen equivalent 20/200 or worse). (**D**) Changes of BCVA in variable degrees of lens subluxation. *: *P* < 0.05.

There was an overall significant improvement of BCVA in1-month follow-up, from 0.64 ± 0.41 logMAR units to 0.24 ± 0.23 logMAR units (Fig. 3A). In the 52 eyes at 3-month follow-ups, significant differences in the BCVA between postoperative 1-month and 3-month were also observed (0.22 ± 0.22 logMAR units, 0.2 ± 0.21 logMAR units, respectively, *P* = 0.028, paired t-tests.). In the 28 eyes at 6-month follow-ups, there was no significant difference in the BCVA between postoperative 3-month and 6-month (0.22 ± 0.24 logMAR units, 0.17 ± 0.18 logMAR units, respectively, *P* = 0.095, Paired t-tests.). MFS patients were divided into two groups by age: the “children” group with 71 eyes (age < 15 years) and the “adults” group with 30 eyes (age ≥ 15 years). There was no significant difference in the BCVA during the first visit and 1-, 3-, and 6-month follow-ups between the “children” group and “adults” group or “mild”, “moderate” and “severe” group (Fig. 3B,D). When differences in the 3 groups of BCVA were analyzed, there was a significant difference of BCVA between the first visit and 1-, 3-month follow-up while no difference was found in the 6-month follow-up (Fig. 3C).

### Linear regression model of BCVA at 1-, 3-, and 6-month Follow-ups

Univariable analysis (Table 3) revealed that sex (P = 0.016) and age (P = 0.007) were significantly associated with BCVA at 1-month follow-up. Specifically, each additional age was associated with an increase of − 0.277 logMAR (95% confidence interval [CI], − 0.008 to − 0.001). Km F, Km TCRP and B/F ratio were associated with deterioration of BCVA at 1-month follow-up (Table 3). The BCVA at 1-,3-,6-month follow-ups started to rise with the increasing of preoperative BCVA (regression coefficient[95%CI]: 0.499[0.155, 0.361], 0.393[0.068, − 0.349], 0.437[0.039, 0.473], respectively).

**Table 3:** Univariate analysis of factors associated with BCVA of the post-operative MFS group.

Baseline variables	1-Month follow-up (N = 101)		3-Month follow-up (N = 52)		6-Month follow-up (N = 28)	
	Beta coefficient (95% CI)	*P* value	Beta coefficient (95% CI)	*P* value	Beta coefficient (95% CI)	*P* value
Sex (female:male)	0.242(0.019, 0.183)	0.016*	0.159(− 0.053, 0.185)	0.217	− 0.09(− 0.172, 0.11)	0.655
Eyes (right:left)	− 0.035(− 0.11, 0.077)	0.728	− 0.176(− 0.187, 0.044)	0.218	0.17(− 0.082, 0.201)	0.396
Age (years)	− 0.277(− 0.008,—0.001)	0.007*	− 0.254(− 0.009, 0)	0.078	− 0.279(− 0.013, 0.002)	0.158
Preoperative BCVA (logMAR)	0.449(0.155, 0.361)	0.001*	0.393(0.068, − 0.349)	0.004*	0.437(0.039, 0.473)	0.023*
Degree of lens dislocation	0.121(0.036, 0.107)	0.326	0.165(− 0.049, 0.141)	0.329	0.117(− 0.001, 0.002)	0.57
Central ECC (cells/mm2)	0.11(0, 0)	0.326	0.195(0, 0)	0.21	0.166(0, 0)	0.471
Corneal pachymetry (μm)	0.117(0, 0.001)	0.316	0.174(− 0.001, 0.002)	0.297	0.17(− 0.001, 0.002)	0.418
WTW (mm)	0.002(− 0.017, 0.018)	0.986	0.225(− 0.046, 0.248)	0.174	0.379(− 0.04, 0.313)	0.121
LT (mm)	− 0.141(− 0.115, − 0.029)	0.237	− 0.301(− 0.185, 0.008)	0.07	− 0.266(− 0.244, 0.081)	0.303
AL (mm)	0.092(− 0.008, 0.022)	0.368	0.072(− 0.013, 0.021)	0.622	− 0.362(− 0.061, 0.002)	0.064
Km F (D)	− 0.285(− 0.066, − 0.011)	0.006*	− 0.186(− 0.058, 0.014)	0.227	0.092(− 0.032, 0.05)	0.663
Astig F (D)	0.036(0.059, − 0.042)	0.734	0.101(− 0.041, 0.082)	0.514	0.306(− 0.016, 0.111)	0.137
Km TCRP (D)	− 0.273(− 0.064, 0.01)	0.009*	− 0.184(− 0.057, 0.014)	0.233	0.14(− 0.027, 0.053)	0.505
Astig TCRP (D)	0.015(− 0.042, 0.048)	0.887	0.083(− 0.041, 0.072)	0.592	0.348(− 0.008, 0.106)	0.088
WFA 4-mm zone (D)	− 0.009(− 0.047, 0.043)	0.933	− 0.106(− 0.082, 0.04)	0.496	0.174(− 0.027, 0.063)	0.416
WFA Z40 (D)	− 0.049(− 0.609, 0.381)	0.649	− 0.169(− 1.169, 0.347)	0.28	0.044(− 0.761, 0.928)	0.835
WFA HO RMS (D)	0.048(− 0.275, 0.435)	0.654	0.093(− 0.683, 1.256)	0.554	0.556(0.514, 2.519)	0.005*
ACD int (mm)	0.179(0.012, − 0.16)	0.092	− 0.091(− 0.177, 0.098)	0.562	− 0.219(− 0.195, 0.064)	0.305
B/F ratio	− 0.233(0.003, 0.057)	0.026*	0.109(− 0.023, 0.048)	0.485	0.297(− 0.013, 0.076)	0.158

BCVA: best-corrected visual acuity; ECC: endothelial cell count; WTW: white to white; LT: lens thickness; AL: Axial length; Km: mean keratometry; F: front (anterior corneal surface); Astig: astigmatism; TCRP: total corneal refractive power; WFA 4-mm zone: wave front aberration in the 4-mm zone around the corneal apex; WFA Z40: total corneal spherical aberrations (Z4,0) in the 6-mm zone around the corneal apex; WFA HO RMS: root mean square of the total corneal high order aberrations calculated in the 4-mm zone around the corneal apex; B/F ratio: mean radius of the posterior corneal surface/mean radius of the anterior corneal surface ratio; ACD: anterior chamber depth; Cornea: corneal diameter (horizontal).

In the multivariable analysis, age, preoperative BCVA, and B/F ratio were significantly associated with 1-month BCVA while preoperative BCVA is the only variable significantly associated with 3-month BCAV (Table 4).

**Table 4:** Multivariate analysis of factors associated with BCVA of the post-operative MFS group.

Baseline variables	Beta coefficient (95% CI)	*P* value
**1-Month follow-up (N = 101)**		
Sex (female:male)	0.083(− 0.209, 0.498)	0.418
Age (years)	− 0.253(− 0.391, − 0.037)	0.019
Preoperative BCVA (logMAR)	0.344(0.13, 0.482)	0.001
Km F (D)	− 0.527(− 1.656, 0.764)	0.465
Km TCRP (D)	0.348(− 0.911, 1.503)	0.627
B/F ratio	0.214(0.014, 0.35)	0.034
**3-Month follow-up (N = 52)**		
Preoperative BCVA (logMAR)	0.393(0.068, 0.346)	0.004
**6-Month follow-up (N = 28)**		
Preoperative BCVA (logMAR)	0.259(− 0.05, 0.301)	0.152
WFA HO RMS (D)	0.52(0.429, 2.405)	0.007

CI: confidence interval; BCVA: best-corrected visual acuity; Km: mean keratometry; F: front (anterior corneal surface); Astig: astigmatism; TCRP: total corneal refractive power; WFA HO RMS: root mean square of the total corneal high order aberrations calculated in the 4-mm zone around the corneal apex; B/F ratio: mean radius of the posterior corneal surface/mean radius of the anterior corneal surface ratio.

## Discussion

EL is a consistent and typical manifestation in approximately 60% of MFS^18^ patients as a result of zonular dialysis^9^. The deficiency of FBN-1 results in challenges in ocular surgeries because of intra-operative and post-operative complications such as anterior capsule tear and RD^1,4^.

Patients with EL may present primarily with fluctuating vision, blurred vision, or monocular diplopia^19^. Prescribing spectacles or contact lenses for MFS patients with lens subluxation may be futile. In the presence of lens subluxation, the overarching goal is to optimally address lenticular astigmatism through a surgical approach^20^. When the lens loses significant zonular support, performing lens extraction with pars plana vitreolensectomy in MFS patients could be challenging and complicated by endothelial cell damage, the loss of the capsular bag, and vitreous disturbance^21^. Alternatively, the within-the-bag lensectomy approach may achieve good visual outcomes with an acceptable complication rate of RD (4.5%)^22^. Besides, the capsular tension ring (CTR) has been commonly recognized as a rewarding and effective procedure in localizing zonular weakness. However, it does not provide accurate decentration of the capsular bag in the presence of progressive zonular dialysis in MFS patients, whereby further decentration or even dislocation of the IOL-CTR-Bag Complex Subluxation may be encountered^23^.

MCTR has been recognized as an excellent device in providing good stabilization of capsular bags with zonular dialysis. As MFS represents a progressive disease with advancing age, MCTR implantation has been chosen for MFS patients in this study. Our study has shown that MCTR preserved the capsular bags safely in MFS patients and consistent surgical outcomes were observed.

In our study group, over 98% of eyes showed an improvement in BCVA at 1-month while the increase of BCVA slowed down after 3-month follow-ups, and tended to be stable after 3 months as no significant difference was observed between the 3- and 6-month follow-ups. The treatment of PCO and amblyopia are essential to achieve good visual acuity. In our cohort, the treatment of amblyopia that gradually improved vision acuity was recommended one month after surgery. Conversely, PCO that developed after surgery might have negated the improved visual acuity brought by amblyopia therapy and finally contributed to the stable BCVA at 3- to 6-month follow-ups.

Our analysis has found a negative correlation between age and BCVA (logMAR units) at 1-month follow-up. Univariable and multivariable line regression analyses revealed that with the additional age, BCVA (logMAR units) at 1-month follow-up would decrease, which suggested that older patients achieved a better visual outcome. Severe amblyopia is more common in MFS children with EL than the individuals who developed EL in adulthood, which may result in poorer visual prognosis in MFS children of whom with possible inherent severe phenotypes than the adults. Meanwhile, a significant difference was observed in BCVA between the first visit and 1-, 3-, and 6-month follow-ups, which indicated that better preoperative BCVA was correlated with better visual outcomes after implantation of MCTR. Although there was no significant correlation between degrees of lens subluxation and BCVA after surgery, a significant correlation was demonstrated in our analysis between degrees of lens subluxation and surgery time. These correlation results suggest that implantation of MCTR should be considered early especially when an EL has occurred.

In our study, the majority (91.81%, 101 eyes) had MCTR implantation with IOL while transscleral suture-fixated IOL was performed in 9 eyes (8.18%) as a result of intra-operative complications. The most common intra-operative complications that occurred were a peripheral extension of the tear and prolapse of vitreous, which were the main reasons for not proceeding with MCTR implantation but a transscleral suture-fixated IOL was performed instead. Significant differences were observed between the varying degrees of lens subluxation and risks of prolapse of vitreous and peripheral extension of the tear, which suggested that patients with severe lens subluxation are at high risk of surgical failure. Moreover, the severity progression of EL was associated with increased surgical time, indicating the increased operative difficulty that further enhancing the risk of intraoperative complications such as a peripheral extension of the tear. Although ideal BCVA was achieved in the 9 eyes operated with transscleral suture-fixated IOL, the risk of postoperative complications also increased. Therefore, these results have further emphasized that in the EL with rapidly deteriorating BCVA, the corrective operation should be expedited to reduce the risk of potential intra-operative and postoperative complications.

Some postoperative complications have been observed during the follow-ups in our cohort, with PCO being the most common complication observed in 21.36% of eyes. Previous studies^24,25^ have reported a high risk of PCO of 60%–84% from a few months to a few years following CTR implantation in children with EL. In our study, posterior capsular opacification was observed in 22 eyes, and ACO was found in 11 eyes (10.68%) at 6-month follow-up, and 9 (93.9%) of these needed an anterior capsulotomy by YAG laser as a precautionary measure in our practice. Comparatively, the lower rates of PCO in our study might be due to the shorter length of follow-up. Retinal detachment is another potential vision-threatening complication of MFS. Without appropriate intervention, the risks of 5–11% in retinal detachments have been reported in MFS patients, with particularly higher risk (8–38%) among those with prior intraocular surgery^11,17^. In this context, MCTR minimizes pulling forces to the vitreous base especially crucial in the setting of MFS. In our study, RD was only observed in 2 MFS patients (1.94%) postoperatively, which is lower than reported in the literature.

There were some limitations to this study. Firstly, it was limited by a fixed, relatively small sample size and rather short and variable follow-ups due to the COVID-19 pandemic in 2020. Secondly, the retrospective methodology and the lack of a control group for comparison might have resulted in biases in our findings. Despite these, our finding of a significant association between the varying degrees of lens subluxation and intro-operative complications lays the foundation for our future study, in addition to a longitudinal study for long-term postoperative complications.

In conclusion, MCTR implantation contributes to an obvious improvement in BCVA. The preoperative BCVA and age are predictors of BCVA outcome postoperatively. The severity of EL determines the length of surgical time and risks of intraoperative complications, with ACO and PCO being the main postoperative complications in the short-term follow-up. Therefore, early operation with MCTR is safe and necessary for MFS patients with EL.

## Data Availability

Some or all data, models, or code generated or used during the study are available from the corresponding author by request.
